# ﻿An updated list of butterflies (Lepidoptera, Papilionoidea) of two Guatemalan seasonally dry forests

**DOI:** 10.3897/zookeys.1118.85810

**Published:** 2022-08-17

**Authors:** Jiichiro Yoshimoto, José Luis Salinas-Gutiérrez, Mercedes Barrios, Andrew D. Warren

**Affiliations:** 1 Laboratorio de Entomología Sistemática, Centro de Estudios Ambientales y Biodiversidad, Universidad del Valle de Guatemala, Apartado Postal 82, 01901, Guatemala City, Guatemala Universidad del Valle de Guatemala Guatemala City Guatemala; 2 Departamento de Biología Evolutiva, Facultad de Ciencias, Universidad Nacional Autónoma de México, Apartado Postal 70–399, México 04510 DF, Mexico Universidad Nacional Autónoma de México Mexico City Mexico; 3 Centro de Estudios Conservacionistas (CECON), Universidad de San Carlos de Guatemala, Avenida La Reforma 0-53, Zona 10, Guatemala City, Guatemala Universidad de San Carlos de Guatemala Guatemala City Guatemala; 4 McGuire Center for Lepidoptera and Biodiversity, Florida Museum of Natural History, University of Florida, 3215 Hull Rd., UF Cultural Plaza, Gainesville, Florida 32611-2710, USA University of Florida Gainsville United States of America

**Keywords:** Annotated list, dissimilarity, Hesperiidae, inventory, Mesoamerica, Neotropics, seasonality

## Abstract

Guatemala has a great diversity of butterflies, although there have been few intensive surveys on Lepidoptera in the country so far. We present an updated list of 218 species in 149 genera, 19 subfamilies, and six families of butterflies sampled at two seasonally dry forests in the Salamá and Motagua valleys in central and eastern Guatemala, by integrating new data from field surveys conducted in 2014–2021 into our previously published data (Yoshimoto et al. 2018, 2019), with *Amblyscirteselissaelissa* Godman, 1900, *Repensflorus* (Godman, 1900), and *Niconiadesnikko* Hayward, 1948 (Hesperiidae: Hesperiinae) as new country records. We collected a hairstreak species, *Chalybshassan* (Stoll, 1790) (Lycaenidae: Theclinae), at the Motagua Valley site, representing the second record for Guatemala since the early 20^th^ century, after we rediscovered it at the Salamá Valley site in 2011 and 2012 (Yoshimoto and Salinas-Gutiérrez 2015). Nymphalidae and Hesperiidae had larger numbers of species than the other four families at both sites. In Pieridae and Nymphalidae, species composition was similar between the sites, whereas in Lycaenidae, Riodinidae, and Papilionidae it differed more greatly between the sites. These results confirm the relatively high lepidopteran diversity of Guatemalan dry forests, noteworthy for the small areas that comprise the study sites, and represent marked similarities and differences in butterfly fauna and phenology within these forests.

## ﻿Introduction

Neotropical seasonally dry forests are rich in flora and fauna (Pennington et al. 2006; Dirzo et al. 2011), although their ecosystems have been deteriorating because of various anthropogenic disturbances, such as deforestation due to agricultural expansion (e.g., Chazdon et al. 2011). Dry forests in Guatemala also harbor high lepidopteran diversity as well; our previous studies documented more than 150 and 100 butterfly species at the two small forest reserves in central and eastern Guatemala, respectively (Yoshimoto et al. 2018, 2019). These species lists, however, are still incomplete, and obviously, more species remain to be sampled at these sites. Moreover, we detected marked seasonal patterns in butterfly species richness and several conspicuous differences in the lepidopteran fauna between the two sites (Yoshimoto et al. 2019). Thus, it was apparent that additional field surveys were needed to make quantitative between-site comparisons of species composition, in order to enhance our understanding of butterfly fauna and phenology of these forests.

In Guatemala, approximately 400 species of Hesperiidae and nearly 700 species of the remaining families of Papilionoidea have been reported (Austin et al. 1998; Barrios et al. 2006; Salinas-Gutiérrez et al. 2009, 2012; Salinas-Gutiérrez 2013). Despite such high lepidopteran diversity, Guatemala’s butterfly fauna has been studied less intensively compared to neighboring countries; for example, in Mexico, exhaustive species lists for the whole country and for several states have been published (de la Maza et al. 1989, 1991; Luis-Martínez et al. 2011, 2016; Llorente-Bousquets et al. 2014), whereas Guatemala has had little research on Lepidoptera and few published inventories since the 20^th^ century (but see Austin et al. 1996 and Yoshimoto et al. 2021). Continued field surveys in various parts of Guatemala are thus important to fill a gap in our knowledge of the Neotropical butterfly fauna, which will in turn contribute to biodiversity conservation in the country.

Here, we present an updated and integrated list of papilionoid species (including Hesperiidae; van Nieukerken et al. 2011) for the same dry forest sites where we conducted our previous studies (Yoshimoto et al. 2018, 2019), by adding the new data from subsequent field surveys performed in 2014–2021, correcting identification errors, and modifying some of the species names based on taxonomic changes. Additionally, we examine between-site differences in butterfly fauna by comparing species composition at the family level, and identify seasonal patterns at the species level.

## ﻿Materials and methods

**Figure 1.**
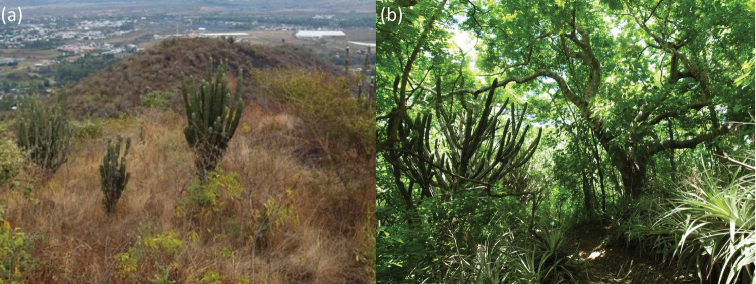
**a** Forest landscape of Los Cerritos Municipal Park and **b**Heloderma Natural Reserve.

This study was conducted at the Los Cerritos Municipal Park (hereafter, Los Cerritos; Fig. 1a) in the Salamá Valley (a subwatershed of the Chixoy region) of Baja Verapaz Department in central Guatemala (15°05'N, 90°18'W, 960–1160 m a.s.l., 69 ha), and at the Heloderma Natural Reserve (hereafter, Heloderma Reserve; Fig. 1b) in the Motagua Valley of Zacapa Department in eastern Guatemala (14°53'N, 89°47'W, 510–790 m a.s.l., 58 ha). The rainfall patterns are similar between the two areas, in which the rainy season usually begins in late May and ends in October; these six months were accordingly defined as the rainy season and the remaining months (November-April) as the dry season. This climatic trait fits the definition of seasonally dry tropical forests (4–6 months with rainfall being < 100 mm; Dirzo et al. 2011); see fig. 1 in Yoshimoto et al. (2018) and fig. 3 in Yoshimoto et al. (2019) for detailed precipitation information of each area.

The vegetation of both regions is characterized by an abundance of various aculeate plants such as cacti (Cactaceae). The most dominant species is a columnar cactus *Stenocereuspruinosus* (Otto) Buxb., with *Pilosocereusleucocephalus* (Poselg.) Byles & G. D. Rowley and *Pereskialychnidiflora* DC., also being abundant at both sites. On the other hand, there exist some marked differences in flora and in forest landscape. Heloderma Reserve has a dense forest with many arboreal species such as *Bucidamacrostachya* Standl. (Combretaceae), *Lysilomadivaricatum* (Jacq.) J. F. Macbr., *Leucaenacollinsii* Britton & Rose (both Mimosaceae), and *Burseraexcelsa* (Kunth) Engl. (Burseraceae), all of which can grow taller than the columnar cactus (Ariano-Sánchez and Salazar 2015; D. Ariano-Sánchez, pers. comm.; Fig. 1b). By contrast, none of these species have been reported from Los Cerritos (M. R. Álvarez, pers. comm.), where there are fewer high arboreal species and abundant shrubs and herbaceous plants (thus commonly called a spiny bush or scrub), thereby the columnar cactus being prominent in its forest landscape (Fig. 1a).

Field surveys were conducted on 21 days from July 2014 to August 2021 at Los Cerritos, and on 19 days from October 2017 to November 2021 at Heloderma Reserve. We collected adult butterflies with an insect net or photographed them in the daytime (09:00–17:00) at each site and in neighboring areas (a small garden at the foot of Los Cerritos and on a farm road adjacent to Heloderma Reserve). The individuals collected were mounted as voucher specimens and were deposited at the Colección de Artrópodos, Laboratorio de Entomología Sistemática, Universidad del Valle de Guatemala. All the individuals collected or photographed were identified to species or subspecies according to Warren et al. (2017). We did not include data for specimens that were not identified to species, except for *Calephelis* spp. (Riodinidae) and *Bolla* sp. (Hesperiidae: Pyrginae); see the footnotes of the Appendix 1 for the rationales for the inclusion of these data. We added all these data to our previous data (Yoshimoto et al. 2018, 2019), corrected identification errors, and modified scientific names of some of the species based on taxonomic changes, in order to compile an updated and integrated species list of the two sites. Note that the sampling methods were partially different in the previous surveys; only netting was done at Los Cerritos from January 2011 to November 2012, whereas at Heloderma Reserve between February 2016 and March 2017, data were obtained through netting, photographing, and observation; see Yoshimoto et al. (2018, 2019) for detailed information on the sampling methods for each site.

The site-level estimated species richness was calculated by using the Chao II index (Chao et al. 2005; Gotelli and Colwell 2011), after pooling the data across observation dates for each month for each site. The total estimated species richness was similarly obtained after pooling these data across both sites. The Jaccard dissimilarity index was used to quantify the between-site similarity in species composition; this index was calculated for all data and for each of the six families (Papilionidae, Pieridae, Lycaenidae, Riodinidae, Nymphalidae, and Hesperiidae). All the analyses were performed using R 4.1.2. (R Development Core Team 2021) with the package Vegan (Oksanen et al. 2020).

## ﻿Results

**Figure 2.**
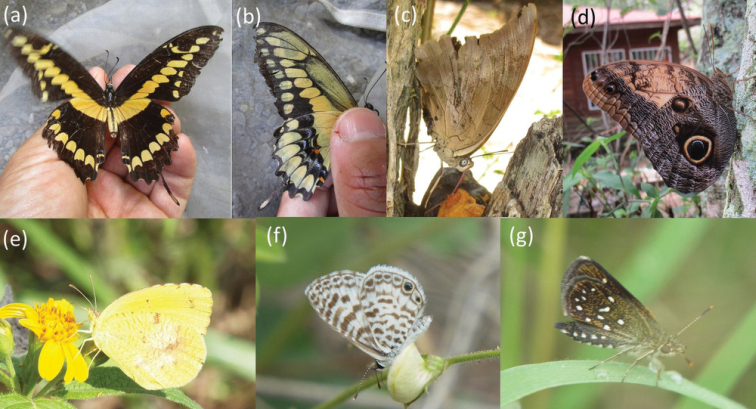
Six of the species that were newly recorded in the present study at Los Cerritos or Heloderma Reserve **a, b***Heraclidesrumiko* Shiraiwa & Grishin, 2014 (Papilionidae) **c***Archaeopreponademophoncentralis* (Fruhstorfer, 1905) **d***Caligotelamoniusmemnon* (C. Felder & R. Felder, 1867) (both Nymphalidae) **e***Abaeisnicippe* (Cramer, 1779) (Pieridae) **f***Leptotescassiuscassidula* (Boisduval, 1870) (Lycaenidae) **g***Pirunaaea* (Dyar, 1912) (Hesperiidae) **a–d, g**Heloderma Reserve **e, f** Los Cerritos. Note that *P.aea* had already been collected and identified to genus (*Piruna* sp.1) by Yoshimoto et al. (2019).

By integrating our previous data (Yoshimoto et al. 2018, 2019), a total of 218 species (including one unidentified taxon and 107 subspecies) in 149 genera from 19 subfamilies of six families were recorded at the two sites (Appendix 1). Hesperiidae was the richest family (71 species), followed by Nymphalidae, Lycaenidae, Pieridae, Riodinidae, and Papilionidae (66, 36, 20, 16, and 9 species, respectively). Los Cerritos had 166 species in 117 genera, and Heloderma Reserve had 139 species in 107 genera (Appendix 1), 16 and 41 species of which had been newly recorded in the subsequent surveys, respectively (Fig. 2). The estimated species richness (mean±SE) of each site based on the Chao II index is 216.27±16.35 and 187.35±17.74, respectively, indicating that approximately 76.8% and 74.2% of the species inhabiting each site were sampled. The total estimated species richness for both sites is 272.80±17.85 (79.9%).

We detected identification errors for 20 individuals (identified as 11 species in our previous studies) and determined them to represent 13 species in this study; ten individuals from Los Cerritos and ten from Heloderma Reserve have been determined to number eight and six different species, respectively, with one species, *Cissiathemis*, shared between sites (Table 1). Additionally, Yoshimoto et al. (2019) incorrectly listed *Piruna* (Hesperiidae: Heteropterinae) in the subfamily Hesperiinae.

**Table 1.** Butterfly species that were sampled at Los Cerritos and Heloderma Reserve (abbreviated as LC and HR, respectively) and were misidentified in Yoshimoto et al. (2018, 2019). Corrected species names are shown in bold.

Family	Species		Sampling month, year, and site
	Correct identification	Previous identification	
Papilionidae	***Heraclidesrumiko* Shiraiwa & Grishin, 2014**	*Heraclidescresphontes* (Cramer, 1777) ^A^	Oct 2016 HR*
Pieridae	***Abaeisnicippe* (Cramer, 1779)**	*Pyrisitiaproterpia* (Fabricius, 1775) ^A^	Jul 2016 HR
Lycaenidae	***Strymonmegarus* (Godart, [1824])**	*Strymonmelinusfranki* W. D. Field, 1938 ^A^	Oct 2016 HR
Nymphalidae	***Anthanassatulcis* (H. Bates, 1864)**	*Anthanassadracaenaphlegias* (Godman, 1901) ^B^	May 2011 LC
Nymphalidae	***Chlosyneerodyleerodyle* (H. Bates, 1864)**	*Chlosynelacinialacinia* (Geyer, 1837) ^B^	Oct 2011 LC, Jul 2012 LC
Nymphalidae	***Chlosynerositarosita* A. Hall, 1924**	*Chlosynelacinialacinia* (Geyer, 1837) ^B^	Sep 2011 LC
Nymphalidae	***Cissiasimilis* (A. Butler, 1867)**	*Cissiapompilia* (C. Felder & R. Felder, 1867) ^B^	May 2012 LC, Jun 2012 LC
Nymphalidae	***Cissiathemis* (A. Butler, 1867)**	*Cissiapompilia* (C. Felder & R. Felder, 1867) ^A, B^	Aug 2011 LC, Aug 2016 HR**, Oct 2016 HR
Hesperiidae	***Urbanusviterboana* (Ehrmann, 1907)**	*Urbanusproteusproteus* (Linnaeus, 1758) ^B^	Nov 2011 LC
Hesperiidae	***Heliopetesmacairamacaira* (Reakirt, [1867])**	*Heliopyrgusdomicelladomicella* (Erichson, [1849]) ^B^	Jul 2012 LC
Hesperiidae	***Amblyscirteselissaelissa* Godman, 1900**	*Piruna* sp.1 ^A^	Aug 2016 HR, Sep 2016 HR
Hesperiidae	***Copaeodesaurantiaca* (Hewitson, 1868)**	*Ancyloxyphaarene* (W. H. Edwads, 1871) ^B^	Mar 2011 LC
Hesperiidae	***Cymaenestrebius* (Mabille, 1891)**	*Cymaenestripunctustheogenis* (Capronnier, 1874) ^A^	Sep 2016 HR**

^A^ Listed in Yoshimoto et al. (2019). ^B^ Listed in Yoshimoto et al. (2018). *Specimen not collected (recorded only by photographing; see Fig. 2a, b for its images). **Two individuals collected.

**Figure 3.**
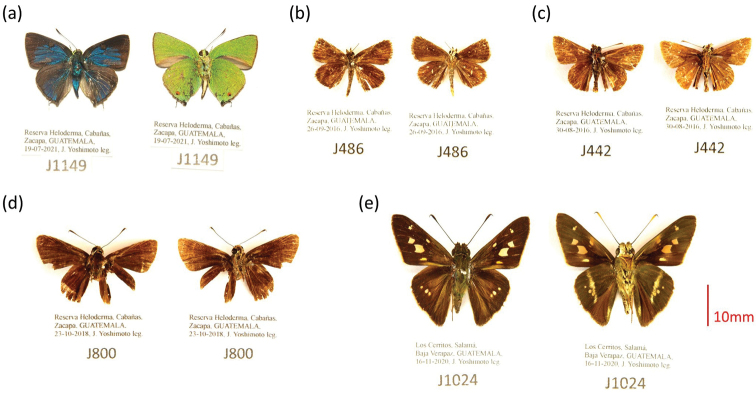
One species of hairstreak (Lycaenidae: Theclinae) **a***Chalybshassan* (Stoll, 1790), one species of skipperling (Hesperiidae: Heteropterinae) **b***Pirunaaea* (Dyar, 1912), and three species of grass-skippers (Hesperiidae: Hesperiinae) **c***Amblyscirteselissaelissa* Godman, 1900 **d***Repensflorus* (Godman, 1900), and **e***Niconiadesnikko* Hayward, 1948. The three grass-skipper species were newly recorded for Guatemala. Dorsal and ventral views, respectively, are shown at the left and right in each photograph.

The following three skipper species (Hesperiidae: Hesperiinae) were recorded for the first time in Guatemala:

- ***Amblyscirteselissaelissa* Godman, 1900.** Reserva Heloderma, Cabañas, Zacapa, GUATEMALA. Three specimens: 30-08-2016, J442; 26-09-2016, J478; 01-06-2018, J769. Collected by Jiichiro Yoshimoto. Identified by Andrew D. Warren. Note that the two individuals (J442 and J478) were misidentified as *Piruna* sp.1 in Yoshimoto et al. (2019), as shown in Table 1. The specimens were deposited in the Colección de Artrópodos, Laboratorio de Entomología Sistemática, Universidad del Valle de Guatemala, and are being cataloged (Fig. 3c). Distribution: Southwestern Mexico (Warren et al. 2017).
- ***Repensflorus* (Godman, 1900).** Reserva Heloderma, Cabañas, Zacapa, GUATEMALA. One specimen: 23-10-2018, J800. Collected by Jiichiro Yoshimoto. Identified by Andrew D. Warren. The specimen was deposited as above and is being cataloged (Fig. 3d). Distribution: Eastern and Western Mexico, Belize, and Nicaragua (Warren et al. 2017).
- ***Niconiadesnikko* Hayward, 1948.** Los Cerritos, Salamá, Baja Verapaz, GUATEMALA. One specimen: 16-11-2020, J1024. Collected and identified by Jiichiro Yoshimoto. The specimen was deposited as above and is being cataloged (Fig. 3e). Distribution: Eastern Mexico to Ecuador, Southern Brazil, and Paraguay (Warren et al. 2017).

**Figure 4.**
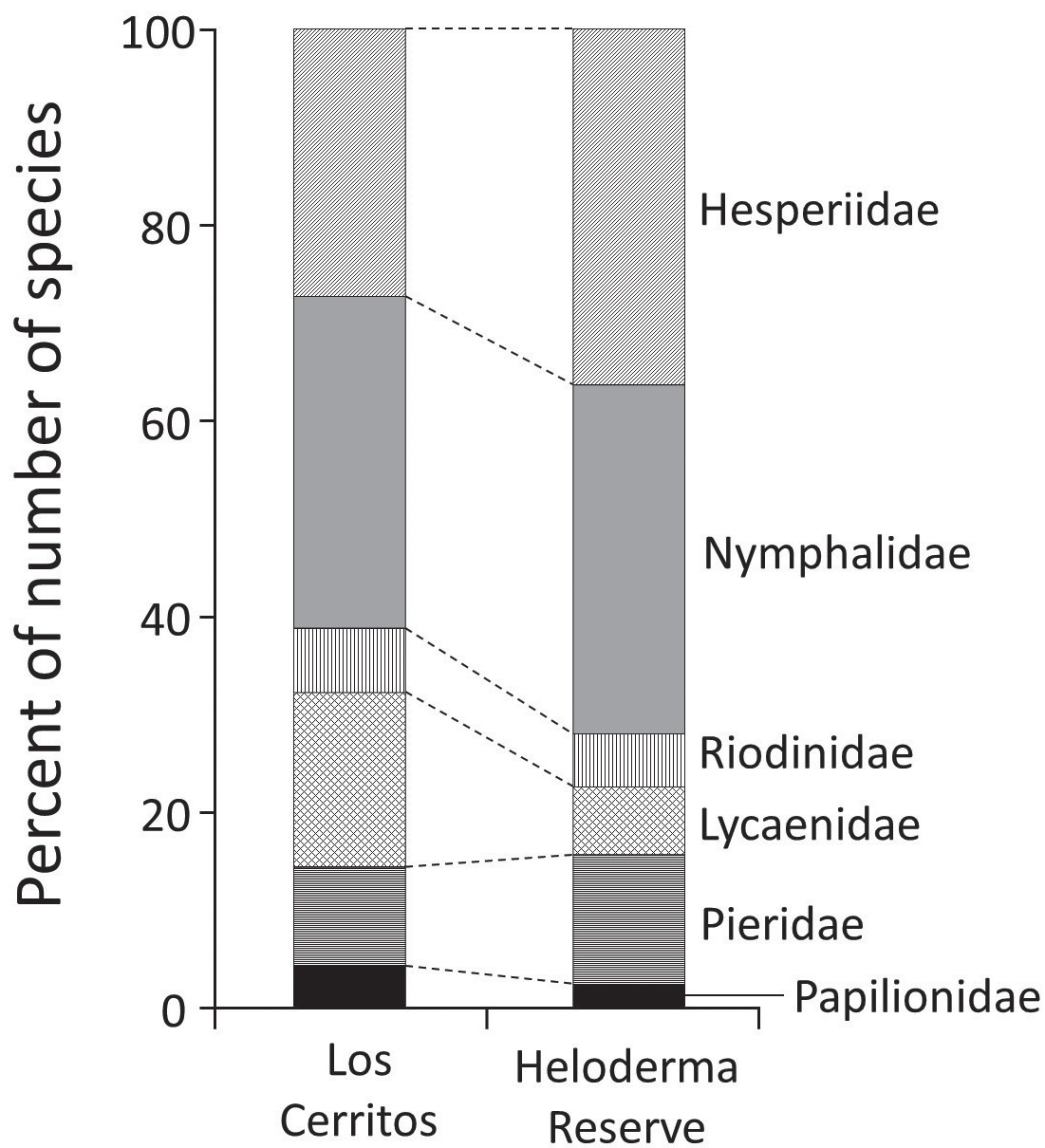
Proportion of species richness at the family level at Los Cerritos and Heloderma Reserve.

Eighty-six species were shared between Los Cerritos and Heloderma Reserve (Table 2), which amounts to 51.8% and 61.9% of the species sampled at each site (the Jaccard dissimilarity index is 0.606). At both sites, species richness of Nymphalidae and Hesperiidae was greater than that of the other four families, although family-level species richness differed greatly between the sites (Table 2; Fig. 4). In particular, the proportion of Lycaenidae was much higher at Los Cerritos (18.7%) than at Heloderma Reserve (8.6%), which was mainly due to differences in the subfamily Theclinae (26 and 7 species, respectively: Appendix 1).

Family-level species composition also differed between the sites, and the magnitude of this difference varied among the six families (Table 2). The dissimilarity indices for Riodinidae, Lycaenidae, and Papilionidae were considerably larger, indicating that species composition differed more greatly between the sites in these families. Pieridae and Nymphalidae, by contrast, had smaller indices with many shared species, demonstrating that their species composition was relatively similar between the sites.

**Table 2.** Species richness for six families at Los Cerritos and Heloderma Reserve, and comparisons of species composition at the family level between the sites, based on the number of shared species and the Jaccard dissimilarity index.

Family	Total No. species		No. shared species	Jaccard index
	Los Cerritos	Heloderma Reserve		
Papilionidae	7	4	2	0.778
Pieridae	16	17	13	0.350
Lycaenidae	31	12	7	0.806
Riodinidae	10	9	3	0.813
Nymphalidae	57	46	37	0.439
Hesperiidae	45	51*	24	0.662*

*The data for *Bolla* sp. were included in the species count but excluded from the Jaccard index analysis (see the footnote 7 of the Appendix 1 for its rationale).

Ninety-three species (42.7%) occurred in both dry and rainy seasons, whereas 103 (47.2%) appeared only in the rainy season and 22 species (10.1%) only in the dry season. The most frequently recorded species was *Euremadairaeugenia* (Wallengren, 1860) (Pieridae: Coliadinae), which was collected or observed throughout the year (Appendix 1). The second most frequently recorded species (in 11 months) were *Kricogonialyside* (Godart, 1819) (Coliadinae) and *Hamadryasglauconomeglauconome* (H. Bates, 1864) (Nymphalidae: Biblidinae), followed by *Pyrisitiaproterpia* (Fabricius, 1775) (Coliadinae: in ten months), *Phoebissennaemarcellina* (Cramer, 1777) (Coliadinae), *Mestraamymone* (Ménétriés, 1857) (Biblidinae), and *Urbanusdorantesdorantes* (Stoll, 1790) (Hesperiidae: Eudaminae: all in nine months).

## ﻿Discussion

A total of 218 species were recorded at the two dry forest sites during our 10-year field surveys, which confirms the relatively high lepidopteran diversity of Guatemalan seasonally dry forests for the small areas that comprise the study sites (<70 ha each). The estimated species richness suggests that nearly a quarter of the species inhabiting each site have yet to been recorded. The number of the additional species yielded in the subsequent surveys was more than twice greater at Heloderma Reserve than at Los Cerritos. The proportion of newly recorded species was much higher in Lycaenidae and Riodinidae; seven lycaenid species were added to the list for Los Cerritos, and seven lycaenid and four riodinid species were added to that of Heloderma Reserve, which nearly doubled the species richness of each family at this site (six lycaenid and five riodinid species in Yoshimoto et al. 2019). Among these species, the record of *Chalybshassan* (Stoll, 1790) at Heloderma Reserve is highly important (Fig. 3a), as this species had not been reported for more than 100 years in Guatemala before we collected four individuals at Los Cerritos in 2011 and 2012 (Yoshimoto and Salinas-Gutiérrez 2015). These results highlight the importance of continuing butterfly surveys at both sites to create more exhaustive inventories, especially on small and taxonomically difficult taxa such as Lycaenidae and Riodinidae. Moreover, it is important to conduct research in other dry regions (e.g., the Nentón Valley in northwestern Guatemala) and to make quantitative among-site comparisons of species richness and composition as well. All these studies will contribute to a comprehensive understanding of Neotropical butterfly fauna and distribution, and would serve as a scientific baseline for biodiversity conservation in Guatemalan dry regions.

More than half of the species sampled at each site were shared between the sites, suggesting that species composition is partially and moderately similar between Los Cerritos and Heloderma Reserve. Importantly, between-site similarity greatly differed among the six families. Higher similarity in Pieridae (especially in Coliadinae) would likely be associated with the distribution and abundance of their host plants, considering that coliadine larvae mostly feed on fabaceous plants such as *Senna* (e.g., DeVries 1987) and that these plants appear to be abundant at both sites.

In Lycaenidae and Riodinidae, species composition largely differed between the sites; in particular, Theclinae had considerable differences in species richness and composition (Appendix 1). In addition, most of these thecline species tended to be highly seasonal, as 25 out of 30 species were sampled only in the rainy season. In contrast to their marked seasonal pattern, *Strymonmegarus* (Godart, [1824]) and *S.rufofusca* (Hewitson, 1877) occurred frequently also in the dry period at Heloderma Reserve; three and five individuals of each species were collected in both December and January at this site (Appendix 1). It should also be mentioned that *Hechtiaguatemalensis* Mez (Bromeliaceae), one of the dominant bromeliad species at Heloderma Reserve (Fig. 1b), may be a possible foodplant for *S.megarus* at this site, as the larvae of this species are known to feed on bromeliads (Robbins 2010). Examination of abundance and distribution of host- and nectar-plants, as well as of larval and adult feeding behavior in relation to their phenology, would be an initial step to elucidate the bionomics of these species. Such surveys may also identify factors underlying the regional similarity and dissimilarity in the butterfly fauna.

We recorded *Amblyscirteselissaelissa* Godman, 1900, *Repensflorus* (Godman, 1900), and *Niconiadesnikko* Hayward, 1948 (Hesperiidae: Hesperiinae) for the first time in Guatemala (Fig. 3c, d, e). Austin et al. (1998) listed *A.e.elissa* and *N.nikko* as species with a potential distribution in Guatemala. *Repensflorus* could have been included in this category as well, as it is known to be distributed in the adjacent countries (Mexico, Belize, and Nicaragua; Warren et al. 2017). These results indicate that there still exists a gap in our knowledge of geographic distribution of Neotropical skipper species, again emphasizing the importance of more intensive research in Guatemala to bridge this gap.

Four individuals of *Pirunaaea* (Dyar, 1912) (two in the previous survey and two in the subsequent one: Figs 2g, 3b) were collected at Heloderma Reserve. This is an interesting result, since most species in this genus are distributed in humid areas at higher elevation (1000–2700 m; Warren and González-Cota 1998). As Yoshimoto et al. (2019) pointed out, the wing pattern of these individuals is somewhat different from Mexican *P.a.aea* (Dyar, 1912), implying that *Pirunacingosombra* Evans, 1955, described from Guatemala and currently considered a synonym of *P.a.aea*, may be a valid subspecies-level taxon. At present, this is difficult to determine, as very few specimens of this species have been sampled in Guatemala (Barrios et al. 2006).

## ﻿Appendix 1

**Table A1.** Butterfly species observed in 2011–2021 at two dry forests in Guatemala: Los Cerritos Municipal Park and Heloderma Natural Reserve, based on our previous studies (Yoshimoto et al. 2018, 2019) and on subsequent field surveys (July 2014 to August 2021 at Los Cerritos and October 2017 to November 2021 at Heloderma Reserve). Species and months in bold indicate the data newly obtained in the subsequent surveys. Year information is also shown with sampling months, when necessary. Nomenclature follows Warren et al. (2017).

Family		Months when observed	
Subfamily		Los Cerritos	Heloderma Reserve
Species and subspecies			
Papilionidae			
Papilioninae			
1	*Neographiumepidausepidaus* (E. Doubleday, 1846) ^PH, A^	Apr, May, **Jun**, **Jul**, **Aug**, **Nov**	–
2	*Neographiumphilolausphilolaus* (Boisduval, 1836) ^PH, A^	Jun, Sep	Mar, Apr, May, Jun
3	*Battuspolydamaspolydamas* (Linnaeus, 1758) ^PH, A^	Mar, Jul, **Aug**, Sep, **Dec**	–
4	***Paridesphotinus* (E. Doubleday, 1844)**	–	**Sep**
5	*Heraclideserostratuserostratus* (Westwood, 1847) ^A, Y^	May, Oct	–
6	*Heraclidesthoasautocles* (Rothschild & Jordan, 1906) ^PH, A^	Feb, **Mar**, Apr, **Jun**, **Aug**, **Nov**	–
7	*Heraclidesornythionornythion* (Boisduval, 1836) ^PH^	–	May, Jun
8	***Heraclidesrumiko* Shiraiwa & Grishin, 2014** ^MI, PH^	**Jul**	Oct’16^MI^, **Dec**
9	*Papiliopolyxenesasterius* Stoll, 1782 ^PH^	Mar, Apr, May, **Nov**	–
Pieridae			
Coliadinae			
10	*Kricogonialyside* (Godart, 1819) ^PH, A^	Mar	Jan, Feb, Mar, Apr, May, Jun, Jul, Aug, Sep, Nov, Dec
11	*Euremadairaeugenia* (Wallengren, 1860) ^PH, A, Y^	Jan, Feb, Aug, Nov	Jan, Mar, Apr, May, Jun, Jul, Aug, Sep, Oct, Dec
12	*Euremaboisduvaliana* (C. Felder & R. Felder, 1865) ^PH, A^	Feb, Oct, Nov	Jun, Jul, Aug, Sep, Oct, Nov
13	***Abaeisnicippe* (Cramer, 1779)** ^MI, PH^	**Nov**	Jul’16^MI^
14	*Pyrisitiaproterpia* (Fabricius, 1775) ^PH, A^	May, Jun, Jul, Oct, **Dec**	Feb, Apr May, Jun, Jul, Aug, Sep, Oct, Nov
15	*Pyrisitiadinawestwoodi* (Boisduval, 1836) ^PH, A^	–	Jan, Feb, Jun, Oct, Nov, Dec
16	*Pyrisitianisenelphe* (R. Felder, 1869) ^PH, A, Y^	**Jun, Jul**, **Aug**, **Nov**	Jun, Jul, Aug, Sep, Oct, **Nov**, Dec
17	*Zerenecesoniacesonia* (Stoll, 1790) ^PH, Y^	Jun, Aug	Jun, **Jul**
18	*Anteosmaerula* (Fabricius, 1775) ^PH, A^	Jun, **Sep**, Nov	**May**, Jun, Jul, Aug, Sep, Oct
19	*Anteosclorinde* (Godart, [1824]) ^PH, A^	Apr, Jun	Jun, Aug
20	*Phoebissennaemarcellina* (Cramer, 1777) ^PH, A^	Feb, **Mar**, Apr, Jun, Jul, Nov	Mar, May, **Jun**, Jul, Aug, Sep
21	*Phoebisphileaphilea* (Linnaeus, 1763) ^A, Y^	May	Jul
22	*Phoebisargante* ssp. ^A^	May	Jul
23	*Aphrissastatirastatira* (Cramer, 1777) ^A^	Oct	–
Pierinae			
24	*Hesperochariscroceacrocea* H. Bates, 1866	Mar, **Aug**	–
25	*Asciamonustemonuste* (Linnaeus, 1764) ^PH, A, Y^	Feb, Jun	Jun
26	*Ganyrajosephinajosepha* (Salvin & Godman, 1868) ^PH, A^	–	Jan, Oct
27	*Leptophobiaaripaelodia* (Boisduval, 1836) ^Y^	Jan	–
28	*Itaballiademophilecentralis* Joicey & Talbot, 1928	–	Jan
29	*Glutophrissadrusillatenuis* (Lamas, 1981) ^A^	–	Jun, Aug
Lycaenidae			
Theclinae			
30	*Evenusregalis* (Cramer, 1775) ^A^	Sep	–
31	*Atlidesgaumeri* (Godman 1901)	Aug	–
32	*Atlidescarpasia* (Hewitson, 1868) ^A^	Aug	–
33	***Rekoazebina* (Hewitson, 1869)**	**Jun, Sep**	–
34	*Rekoastagira* (Hewitson, 1867) ^A^	Aug	–
35	*Arawacussito* (Boisduval, 1836) ^A, Y^	Aug	–
36	*Arawacusjada* (Hewitson, 1867) ^A^	Jul	–
37	*Kolanalyde* (Godman & Salvin, 1887) ^A^	Sep	–
38	***Chlorostrymonsimaethissarita* (Skinner, 1895)** ^A^	**Nov**	–
39	*Cyanophrysherodotus* (Fabricius, 1793) ^A^	Aug	–
40	*Cyanophrysmiserabilis* (Clench, 1946)	–	Oct
41	***Electrostrymonhugon* (Godart, [1824])**	–	**Jul**
42	***Kisutamsyllis* (Godman & Salvin, 1887)** ^A^	–	**Oct**
43	***Calycopisclarina* (Hewitson, 1874)**	**Jun**	–
44	*Calycopisisobeon* (A. Butler & H. Druce, 1872)	Aug, Sep	–
45	*Strymonmelinusfranki* W. D. Field, 1938	Aug, Sep	–
46	*Strymonrufofusca* (Hewitson, 1877) ^PH^	**Jul**, Nov	**Jan**, Aug, Oct, Nov, **Dec**
47	*Strymonbebrycia* (Hewitson, 1868) ^PH^	**Jun**, Aug	–
48	***Strymonyojoa* (Reakirt, [1867])** ^A^	**Jul**	–
49	*Strymoncestri* (Reakirt, [1867]) ^A^	Aug	–
50	*Strymonbazochiibazochii* (Godart, [1824]) ^A^	Jul	–
51	*Strymonistapaistapa* (Reakirt, [1867])	Aug, **Nov**	–
52	***Strymonmegarus* (Godart, [1824])** ^MI, PH^	–	**Jan**, **Jul**, Oct’16^MI^, **Dec**
53	*Strymonziba* (Hewitson, 1868)	Jul	–
54	***Ministrymonazia* (Hewitson, 1873)** ^A^	**Jun**	**Jul**
55	***Ostrinoteskeila* (Hewitson, 1869)** ^A, Y^	**Aug**	–
56	*Panthiadesbitias* (Cramer, 1777) ^A^	Jun	–
57	*Michaelushecate* (Godman & Salvin, 1887)	Sep	–
58	*Eroragabina* (Godman & Salvin, 1887)	May, Jun, Aug, Oct	–
59	*Chalybshassan* (Stoll, 1790)	Aug, Sep, Nov	**Jul**
Polyommatinae			
60	*Celastrinaechogozora* (Boisduval, 1870) ^Y^	Nov	–
61	***Leptotescassiuscassidula* (Boisduval, 1870)** ^PH, A, Y^	**Jun**, **Dec**	**Sep**, **Oct**
62	*Cupidocomyntastexana* (F. Chermock, 1945) ^PH, A, Y^	**Sep**, Nov	**Oct**, Nov, **Dec**
63	*Hemiargusceraunusastenidas* (Lucas, 1857) ^A^	Mar, **Jul**, **Nov**	Feb, Jun, **Dec**
64	*Hemiargushannohanno* (Stoll, 1790) ^PH, A^	–	Jul, Aug, Sep, Oct
65	*Echinargusisola* (Reakirt, [1867])	Feb, **Dec**	**Jun**, **Dec**
Riodinidae			
Riodininae			
66	*Rhetusarciuscastigatus* Stichel, 1909 ^A^	Sep	–
67	*Calephelis* spp. ^PH, 1^	Jan, May, Jul, Aug, Oct, **Nov**, **Dec**	Jan, Jul, Aug, Sep, Oct, Nov, Dec
68	*Lasaiasulasula* Staudinger, 1888 ^PH^	–	Jun, **Oct**
69	***Lasaiamariamaria* Clench, 1972**	–	**Jun**, **Jul**, **Oct**
70	*Melanispixepixe* (Boisduval, 1836) ^A^	Feb, Sep, Nov, Dec	–
71	*Anteroscarausiuscarausius* Westwood, 1851 ^A^	Aug, Nov	**Sep, Nov**
72	*Calydnasturnula* (Geyer, 1837) ^PH^	Aug, **Sep**, Oct	–
73	*Emesismandana* furor A. Butler & H. Druce, 1872 ^A^	Aug	–
74	*Emesistenedia* C. Felder & R. Felder, 1861 ^A, Y, 2^	Jul	–
75	*Emesislupinalupina* Godman & Salvin, 1886 ^2^	Oct	–
76	*Curvieemesia* (Hewitson, 1867) ^PH, A, 3^	–	Jun, Oct
77	*Thisbelycorias* (Hewitson, [1853]) ^A^	Jun, Jul, Oct, Nov	–
78	***Judithacaucana* (Stichel, 1911)**	–	**Oct**
79	*Synargismycone* (Hewitson, 1865) ^A^	Mar, Jun, Jul	Jul
80	***Hypophyllazeurippa* Boisduval, 1836**	–	**Jan**
81	*Theopevirgilius* (Fabricius, 1793) ^A^	–	Feb, Nov
Nymphalidae			
Libytheinae			
82	*Libytheanacarinentamexicana* Michener, 1943 ^PH, A, Y^	Jul	Jun, Aug, Sep
Danainae			
83	*Lycoreahaliaatergatis* E. Doubleday [1847] * ^PH, A, Y^	–	**May**, Sep
84	*Danauseresimusmontezuma* Talbot, 1943 ^PH, A^	Aug, **Nov**, **Dec**	Jun, Aug, **Sep**
85	*Danausgilippusthersippus* (H. Bates, 1863) ^A^	–	Mar
86	*Mechanitislysimniautemaia* Reakirt, 1866 ^PH, A, Y^	–	**May**, Aug, **Oct**
87	*Mechanitispolymnialycidice* H. Bates, 1864 ^PH, A, Y^	Sep	Sep, **Oct**
88	*Dircennaklugiiklugii* (Geyer, 1837) ^Y^	Sep, Oct, Nov	–
Heliconiinae			
89	*Agraulisvanillaeincarnata* (N. Riley, 1926) ^PH, A^	Jun, Jul, **Nov**	Aug
90	*Dionemonetapoeyii* A. Butler, 1873 ^Y^	Jun, Nov	–
91	*Dionejunohuascuma* (Reakirt, 1866) ^PH, A^	Feb, Mar, **Dec**	Jul
92	*Dryasiuliamoderata* (N. Riley, 1926) ^PH, A, Y^	Aug	Aug, Sep, Oct
93	*Eueidesisabellaeva* (Fabricius, 1793) ^A^	Nov	–
94	*Heliconiuscharithoniavazquezae* W. Comstock & F. Brown, 1950 ^A, Y^	Jul	Jul, Oct
95	*Euptoietahegesiameridiania* Stichel, 1938 ^PH, A, Y^	Jun, Jul, Sep	Jun, Jul
Limenitidinae			
96	*Adelphaparoecaparoeca* (H. Bates, 1864) ^Y^	Oct, Nov	–
97	*Adelphaiphicleolaiphicleola* (H. Bates, 1864) ^PH^	Aug	Jun, Jul, Sep, Oct
98	*Adelphamelanthe* (H. Bates, 1864) ^A^	Aug, Sep	–
Biblidinae			
99	*Biblishyperiaaganisa* Boisduval, 1836 ^PH, A^	Jul, **Dec**	Sep
100	*Mestraamymone* (Ménétriés, 1857) ^A^	May, Jun, Jul	Mar, Jul, Aug, Sep, Oct, Nov, Dec
101	*Catonephelemexicana* Jenkins & R.G. Maza, 1985 ^A^	Sep, Oct, Nov	–
102	*Eunicamonima* (Stoll, 1782) ^PH^	Jun, **Aug**	Mar, Jun, Jul, Aug, Oct, Dec
103	*Eunicatatilatatila* (Herrich-Schäffer, [1855]) ^A^	Jun	–
104	*Hamadryasatlantisatlantis* (H. Bates, 1864) ^PH^	Sep	Jun, Jul, Nov
105	*Hamadryasfebruaferentina* (Godart, [1824]) ^PH, A^	Apr, May, **Jun**, **Jul**, **Nov**, **Dec**	Feb, **Jul**, Oct
106	*Hamadryasglauconomeglauconome* (H. Bates, 1864) ^PH^	Jan, **Jul**, Oct, **Nov**, Dec	Jan, Feb, Mar, **May**, Jun, Jul, Aug, **Sep**, **Oct**
107	*Hamadryasguatemalenaguatemalena* (H. Bates, 1864) ^PH, A^	May, Jun, Jul	**Jul**
108	*Bolboneurasylphissylphis* (H. Bates, 1864) ^PH^	Jul, Aug, Sep	Mar, Jun, Jul, Sep, Oct
109	*Epiphileadrastaadrasta* Hewitson, 1861 ^Y^	Aug, Oct	–
110	*Temenislaothoehondurensis* Fruhstorfer, 1907 ^A^	–	Oct
111	*Dynaminedyonis* Geyer, 1837 ^A^	Jul, Aug, Oct, Nov, **Dec**	–
112	*Dynaminepostvertamexicana* R.F. d’Almeida, 1952 ^PH, A^	Jun, Jul, Oct, **Nov**	Sep, Oct
113	*Dynaminetheseus* (C. Felder & R. Felder, 1861) ^A^	**Aug**, Sep, Oct	–
114	*Diaethriaastalaastala* (Guérin-Méneville, [1844]) ^A, Y^	May, Jul, Oct, Nov	**Oct**
Cyrestinae			
115	*Marpesiapetreus* ssp. ^A, Y^	Jul, Sep	Jun
Nymphalinae			
116	*Historisodiusdious* Lamas, 1995 ^PH, A^	**Jun**, **Jul**, Aug, Sep	Jul
117	*Smyrnablomfildiadatis* Fruhstorfer, 1908 ^PH, A, Y^	Jul	**May**, Nov
118	*Anartiafatimafatima* (Fabricius, 1793) ^PH, A, Y^	May	**Jun**, Sep, Oct
119	*Siproetaepaphusepaphus* (Latreille, [1813]) ^A, Y^	Sep	Sep
120	*Siproetastelenesbiplagiata* (Fruhstorfer, 1907) ^PH, A, Y^	Jul, Sep	**Jun**, Aug, Sep, Oct
121	*Junoniaevarete* (Cramer, 1779) ^PH, A^	Jun, Jul, Aug	**Jan**, **Jul**, **Oct**
122	*Chlosynejanaisjanais* (Drury, 1782) ^A^	Jun, **Aug**	–
123	***Chlosyneerodyleerodyle* (H. Bates, 1864)** ^MI^	Jul’12^MI^, **Jul’19**, Oct’11^MI^, **Oct’17**	–
124	***Chlosynerositarosita* A. Hall, 1924** ^MI^	Sep’11^MI^	**Jun**, **Jul**, **Sep**
125	*Chlosynetheonatheona* (Ménétriés, 1855) ^PH^	Apr, Jun, Sep	Jun, Jul, Aug, Sep
126	*Chlosynelacinialacinia* (Geyer, 1837) ^PH, A, Y^	Mar, **Jun**, **Jul**, Aug, Nov	Jun, Jul, Aug
127	*Chlosynemelanarge* (H. Bates, 1864) ^PH^	–	Aug, **Sep**, Oct
128	*Microtiaelvahorni* Rebel, 1906 ^PH^	Jun, Jul, Aug, Nov	Jun, Jul, Aug, Sep, Oct, Nov
129	***Anthanassatulcis* (H. Bates, 1864)** ^MI, A^	May’11^MI^	**Jun**, **Sep, Dec**
130	*Anthanassaptolycaptolyca* (H. Bates, 1864) ^Y^	Aug	**Dec**
131	*Tegosaguatemalena* (H. Bates, 1864) ^A^	Feb, Nov	–
Charaxinae			
132	*Zaretisellops* (Ménétriés, 1855) ^A^	Jul, Sep, Nov	–
133	*Anaeaaidea* (Guérin-Méneville, [1844]) ^PH, A^	May, Nov	Jun, Aug, Oct, Nov
134	*Fountaineaglyceriumglycerium* (E. Doubleday, [1849]) ^PH^	Aug, Sep, Oct, **Nov**	–
135	***Archaeopreponademophoncentralis* (Fruhstorfer, 1905)** * ^PH, A^	–	**Jul**
Satyrinae			
136	*Morphohelenor* ssp.* ^A^	–	Sep
137	***Caligotelamoniusmemnon* (C. Felder & R. Felder, 1867)** * ^PH, A^	–	**Oct**
138	***Manatariahercynamaculata* (Hopffer, 1874)** ^A, Y^	–	**Jun**
139	*Cissiasimilis* (A. Butler, 1867) ^MI, PH, A^	May’12^MI^, Jun’12^MI^, Oct, Nov	Jan, Feb, Apr, May, Jun, Oct, Nov, Dec
140	*Cissiathemis* (A. Butler, 1867) ^MI, PH^	Jul, Aug’11^MI^	Feb, **Jun**, Jul, Aug’16^MI^, Oct’16^MI^, Dec
141	*Cyllopsisgemmafreemani* (D. Stallings & J. Turner, 1947)	Sep, Nov	–
142	*Cyllopsishedemannihedemanni* R. Felder, 1869 ^Y^	Feb	–
143	*Cyllopsishilaria* (Godman, 1901)	Sep, Nov	–
144	*Cyllopsispephredo* (Godman, 1901) ^Y^	**Jun**, Nov	–
145	*Euptychiafetna* A. Butler, 1870	Aug, Sep	–
146	*Hermeuptychiahermes* (Fabricius, 1775) ^A, Y, 4^	Jul	**Jan**, Feb, Sep, Oct
147	*Taygetisthamyra* (Cramer, 1779) ^PH^	Nov	**Jun**, **Oct**
Hesperiidae			
Eudaminae			
148	***Phocidespolybiuslilea* (Reakirt, [1867])** ^A^	**Nov**	–
149	*Phocidesuraniaurania* (Westwood, 1852)	Aug	–
150	*Proteidesmercuriusmercurius* (Fabricius, 1787) ^PH, A^	Jun	**Jun**, Sep
151	*Epargyreusexadeuscruza* Evans, 1952 ^A, Y^	Feb, Mar, Apr, Jun, **Jul**	Aug
152	*Polygonusleoarizonensis* (Skinner, 1911)	Jul	Jun, Jul, Aug, Sep, Oct
153	*Chioidesalbofasciatus* (Hewitson, 1867) ^A^	Jun	–
154	*Chioideszilpa* (A. Butler, 1872) ^A^	Jan, Mar	–
155	*Typhedanusundulatus* (Hewitson, 1867) ^A^	Feb, Mar, May	–
156	*Typhedanusampyx* (Godman & Salvin, 1893) ^A^	–	Oct
157	*Polythrixasine* (Hewitson, 1867) ^PH, A, 5^	–	**Jan**, **Dec**
158	***Polythrixoctomaculata* (Sepp, [1844])** ^A^	–	**May**
159	*Cephiseaelius* (Plötz, 1880)	–	Oct
160	*Codatractusalcaeusalcaeus* (Hewitson, 1867)	Mar, May	–
161	***Codatractusmelon* (Godman & Salvin, 1893)**	–	**Jun**, **Jul**
162	*Urbanusviterboana* (Ehrmann, 1907) ^MI, A, Y^	Sep, Nov’11^MI^	Sep, Oct
163	*Urbanusesmeraldus* (A. Butler, 1877) ^A, Y^	Aug, Sep	**Jun**
164	*Urbanusdorantesdorantes* (Stoll, 1790) ^PH, A^	May, **Jul**, **Aug**, **Dec**	Apr, Jun, Jul, Aug, Sep, Oct, Nov
165	*Urbanusprocne* (Plötz, 1881) ^PH, A, Y^	May, Jul, **Nov**	–
166	*Urbanusdoryssusdoryssus* (Swainson, 1831) ^A^	Jul	–
167	*Astraptesfulgeratorazul* (Reakirt, [1867]) ^A, Y, 6^	Oct, Nov	–
168	*Astraptesalectorhopfferi* (Plötz, 1881) ^A^	Sep	**Jan**
169	*Astraptesanaphusannetta* Evans, 1952 ^PH, A, Y^	Jun, Oct	Jul
170	*Achalarustoxeus* (Plötz, 1882) ^A^	–	Mar, **Apr**, Oct
171	*Achalarusalbociliatusalbociliatus* (Mabille, 1877) ^A^	Feb, Mar	Oct, Nov
172	*Cabarespotrillopotrillo* (Lucas, 1857) ^A, Y^	–	Jun, **Jul**, Aug, **Oct**, Nov
173	*Cogiacajetaeluina* Godman & Salvin, 1894	May, **Jun**	–
Pyrginae			
174	***Mysoriaaffinis* (Herrich-Schäffer, 1869)**	–	**Oct**
175	*Celaenorrhinusfritzgaertneri* (Bailey, 1880) ^PH^	Feb, Aug	Mar, **Jun**
176	*Noctuanastator* (Godman, 1899) ^PH, A, Y^	Feb, Mar, May, **Jul**, Sep	–
177	*Bollaevippe* (Godman & Salvin, 1896)	Mar	–
(177)	*Bolla* sp. ^7^	–	**Oct** ^7^
178	*Staphylusascalaphus* (Staudinger, 1876) ^Y^	May	Jan, Sep
179	*Staphylusazteca* (Scudder, 1872)	–	Feb, Aug, Nov
180	*Gorgythionvox* Evans, 1953 ^A, Y^	–	Jun, Jul, Aug, Sep, Oct
181	***Mylonsalvia* Evans, 1953**	**Nov**	–
182	***Mylonpelopidas* (Fabricius, 1793)** ^A^	–	**May**, **Jun**
183	*Graisstigmaticusstigmaticus* (Mabille, 1883) ^PH^	–	**Jan**, Jun, Jul
184	*Timocharestrifasciatatrifasciata* (Hewitson, 1868) ^A^	–	Jan
185	*Chiomarageorginageorgina* (Reakirt, 1868)	Jan, **Jul**, Sep, **Nov**	Aug
186	***Erynnisfuneralis* (Scudder & Burgess, 1870)**	**Aug**	**Jun**
187	*Eantistamenund* (W. H. Edwards, 1871) ^PH^	Feb, Jul, Aug, **Nov**, Dec	–
188	*Atarnessallei* (C. Felder & R. Felder, 1867) ^A^	Jul	–
189	***Carrhenesfuscescensfuscescens* (Mabille, 1891)**	–	**Jun**
190	*Antigonuserosus* (Hübner, [1812]) ^PH, A^	**Mar**, Oct	Feb, Mar, **Jun**, Aug, Oct
191	*Antigonuscorrosus* Mabille, 1878 ^A, Y^	–	Sep
192	*Zopyrionsandace* Godman & Salvin, 1896	**Mar**, Aug	**Jan**, Apr, **May**, **Jun**, Sep, Dec
193	*Pyrgusoileus* (Linnaeus, 1767) ^PH, A, Y^	Apr	Jul, Aug, Sep, Oct, Dec
194	***Pyrgusorcus* (Stoll, 1780)**	–	**Jun**
195	*Heliopyrgusdomicelladomicella* (Erichson, [1849])	Sep	Sep, **Oct, Nov**
196	*Heliopeteslavianalaviana* (Hewitson, 1868) ^A^	–	Feb
197	*Heliopetesmacairamacaira* (Reakirt, [1867]) ^MI, A^	Jul’12^MI^, **Nov**	Oct
198	*Heliopetesalana* (Reakirt, 1868) ^A, Y^	Jul	–
Heteropterinae			
199	***Pirunaaea* (Dyar, 1912)** ^PH^	–	Jun ^8^, **Jul**, Sep ^8^, **Oct**
Hesperiinae			
200	*Pericharesadela* (Hewitson, 1867) ^A^	Aug, Sep	Oct
201	*Copaeodesaurantiaca* (Hewitson, 1868) ^MI^	Mar’11^MI^	–
202	***Panoquinalucas* (Fabricius, 1793)** ^A^		**Jan**
203	*Zenisjebushemizona* (Dyar, 1918)	Jan	–
204	*Synapteshiva* Evans, 1955	Aug	Jun, Dec
205	*Synaptesyraces* (Godman, 1901)	–	Sep
206	***Callimormussaturnus* (Herrich-Schäffer, 1869)** ^A^	–	**Oct**
207	***Amblyscirteselissaelissa* Godman, 1900 ^NR^** ^, MI^	–	**Jun**, Aug’16^MI^, Sep’16^MI^
208	*Amblyscirtestoltecatolteca* Scudder, 1872 ^A^	**Jun**, Jul	**May**
209	*Methionopsisina* (Plötz, 1882) ^A^	–	**Jan**, Oct, Nov
210	***Repensflorus* (Godman, 1900) ^NR^**	–	**Oct**
211	*Cymaenestrebius* (Mabille, 1891) ^MI, A^	Aug	Sep’16^MI^
212	*Leremaliris* Evans, 1955	–	Jul, Sep
213	***Niconiadesnikko* Hayward, 1948 ^NR^**	**Nov**	–
214	*Vettiusfantasos* (Cramer, 1780) ^A, Y^	Aug, Sep, Oct	**Jan**, Oct
215	*Hylephilaphyleusphyleus* (Drury, 1773) ^A^	Aug	–
216	*Politesvibexpraeceps* (Scudder, 1872) ^A^	Mar, Apr	–
217	***Pompeiuspompeius* (Latreille, [1824])** ^A, Y^	–	**Jun**
218	*Atrytonopsisovinia* (Hewitson, 1866) ^PH^	Jan, Feb, Mar, Apr, **Oct**	Oct, Dec

^PH^ Species photographed. ^A^ Species reported at Tikal in northern Guatemala by Austin et al. (1996). ^Y^ Species reported at Parque Cayalá in Guatemala City by Yoshimoto et al. (2021). ^*^ Specimens not collected (recorded only by direct observation or photographs). ^MI^ Species misidentified in Yoshimoto et al. (2018 or 2019); their previous identification results are shown in Table 1. ^1^ Listed at the genus level because of the difficulty of species-level identification derived from the confused taxonomic state of this genus. ^2^ These two species, together with *Emesistegula* and *E.toltec*, can be treated as a species complex. These taxa require further study, according to Trujano-Ortega et al. (2021). ^3^ The genus name, treated as *Emesis* in Yoshimoto et al. (2019), was modified according to Zhang et al. (2019). ^4^ The name “*hermes*” is correctly applied to a South American species, according to Cong and Grishin (2014). Thus, the individuals collected might include multiple species, none of which are true *hermes*. ^5^ One sample which was photographed at Heloderma Reserve in October 2016 and was identified as this species in Yoshimoto et al. (2019) was excluded, as we found that it might have been of another species of this genus, which is unable to de determined because of the lack of a specimen. ^6^ This is a species complex, which includes several species in Costa Rica (Hebert et al. 2004; Brower, 2006, 2010). Thus, the individuals collected might also be of multiple species. ^7^ These data were included in the species count and Chao II analyses for Heloderma Reserve, since at this site none of the identified species of this genus had been recorded. In contrast, these data have been excluded from the Chao II analysis for all data (pooled across the sites) and from the Jaccard index analyses, as this individual might be of *Bollaevippe* (Godman & Salvin, 1896), which is unable to be examined because of the heavily damaged specimen of *Bolla* sp. ^8^ Identified to genus (*Piruna* sp.1) and incorrectly listed as Hesperiinae in Yoshimoto et al. (2019). **^NR^** New record for Guatemala.
